# Updates on Mechanisms of Cytochrome P450 Catalysis of Complex Steroid Oxidations

**DOI:** 10.3390/ijms25169020

**Published:** 2024-08-20

**Authors:** F. Peter Guengerich, Yasuhiro Tateishi, Kevin D. McCarty, Francis K. Yoshimoto

**Affiliations:** 1 Department of Biochemistry, Vanderbilt University School of Medicine, Nashville, TN 37232, USA; yasuhiro.tateishi@vanderbilt.edu (Y.T.); kevin.d.mccarty@vanderbilt.edu (K.D.M.); 2 Department of Chemistry, University of Texas at San Antonio, San Antonio, TX 78249, USA; francis.yoshimoto@utsa.edu

**Keywords:** cytochrome P450, steroids, steroid oxidations, carbon-carbon bond cleavage, steroid biosynthesis, oxidation mechanisms, ferric peroxide, processivity, cholesterol, lanosterol

## Abstract

Cytochrome P450 (P450) enzymes dominate steroid metabolism. In general, the simple C-hydroxylation reactions are mechanistically straightforward and are generally agreed to involve a perferryl oxygen species (formally FeO^3+^). Several of the steroid transformations are more complex and involve C-C bond scission. We initiated mechanistic studies with several of these (i.e., 11A1, 17A1, 19A1, and 51A1) and have now established that the dominant modes of catalysis for P450s 19A1 and 51A1 involve a ferric peroxide anion (i.e., Fe^3+^O_2_¯) instead of a perferryl ion complex (FeO^3+^), as demonstrated with ^18^O incorporation studies. P450 17A1 is less clear. The indicated P450 reactions all involve sequential oxidations, and we have explored the processivity of these multi-step reactions. P450 19A1 is distributive, i.e., intermediate products dissociate and reassociate, but P450s 11A1 and 51A1 are highly processive. P450 17A1 shows intermediate processivity, as expected from the release of 17-hydroxysteroids for the biosynthesis of key molecules, and P450 19A1 is very distributive. P450 11B2 catalyzes a processive multi-step oxidation process with the complexity of a chemical closure of an intermediate to a locked lactol form.

## 1. Introduction

Steroid metabolism is common to many forms of life, even complex bacteria such as Mycobacteria [1,2,3,4,5]. In humans, ~1/4 of the 57 cytochrome P450 (P450, CYP) enzymes are involved primarily in steroid metabolism (1B1, 7A1, 7B1, 8B1, 11A1, 11B1, 11B2, 17A1, 19A1, 21A2, 27A1, 39A1, 46A1, and 51A1) [6,7,8,9,10,11,12,13,14,15,16,17,18,19,20]. In addition, many other human P450s are also capable of catalyzing steroid oxidations, even if the process is not considered physiologically critical (e.g., P450s 1A2, 2C9, 3A4, 3A5) [21,22,23,24,25,26]. The P450s in the former group are considered essential, and deficiencies in these genes are problematic, leading to endocrine problems [27,28,29,30,31,32,33,34]. However, several of these P450s are also drug targets in cases where attenuation of the products is desirable [35,36,37,38,39,40,41,42].

A number of the P450-catalyzed steroid oxidations are complex and have attracted interest from enzymologists, not only from a pedantic view but also in the context of drug discovery. In addition, the catalytic mechanisms of these P450 reactions have counterparts in drug metabolism [43,44,45,46] and in the biosynthesis of natural products [47,48,49].

**Figure 1:**
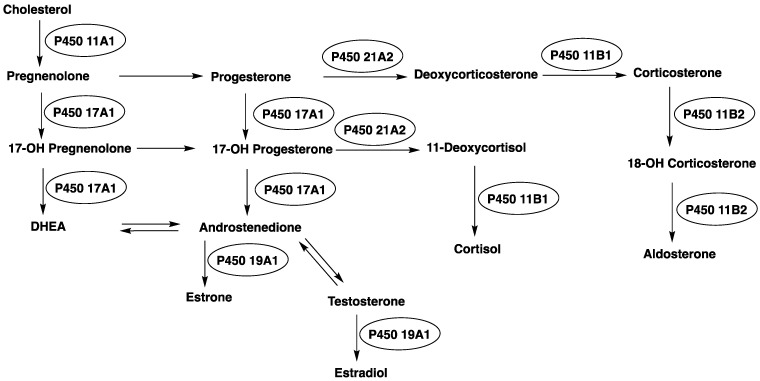
Roles of P450s in mammalian steroid metabolism.

**Figure 2:**
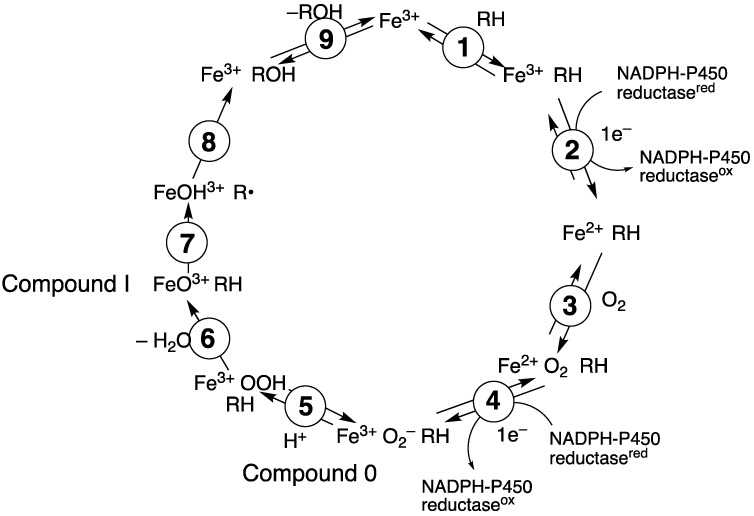
General catalytic cycle for P450 oxidations.

P450 enzymes serve as the main catalysts in steroid oxidations (Figure 1). In the recent past, much of the P450 research in our own laboratory has been directed toward several steroid oxidations, particularly considering the kinetic processivity of multi-step reactions and the roles of different iron-oxygen complexes in catalysis (Figure 2). This review will update some primary literature and also recent reviews from our group [50,51]. Five of these enzymes will be considered here: P450s 11A1, 11B2, 17A1, 19A1, and 51A1.

## 2. P450 11A1

**Figure 3:**
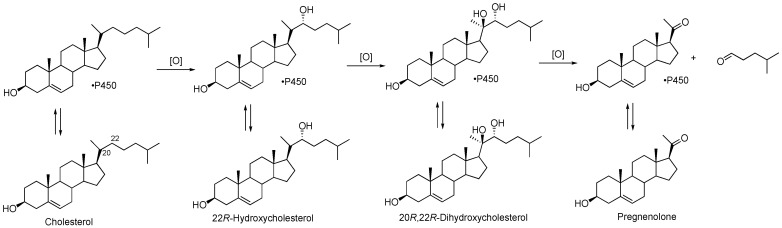
Conversion of cholesterol into pregnenolone by P450 11A1.

P450 11A1 is the classic cholesterol side chain cleavage enzyme (P450_scc_) that converts the sterol cholesterol into other steroids (Figure 3). It also uses other sterols as substrates [52,53], as well as vitamin D [54,55,56,57] and even some drugs [58].

**Figure 4:**
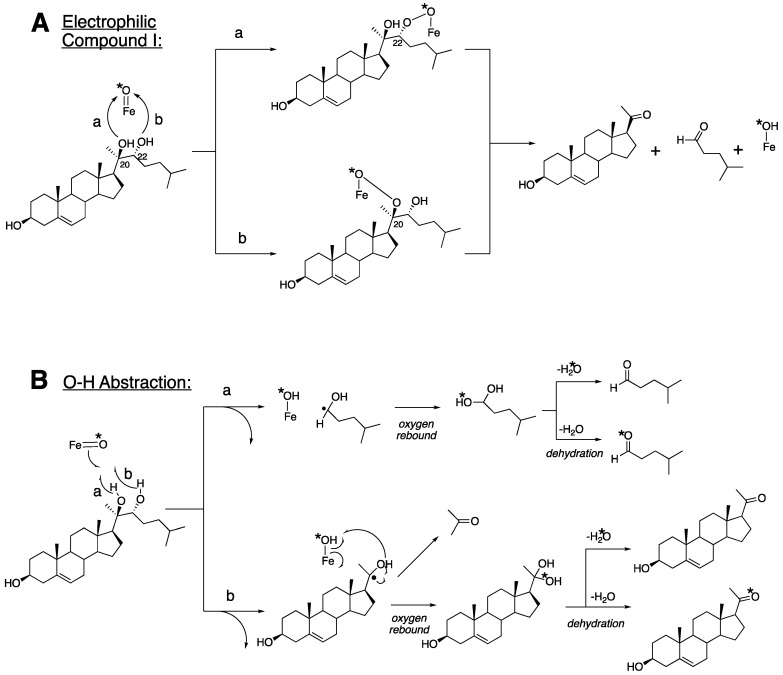
Proposed mechanisms of the C-C bond cleavage step for P450 11A1 [63]. The alternate pathways a and b are shown.

**Figure 5:**
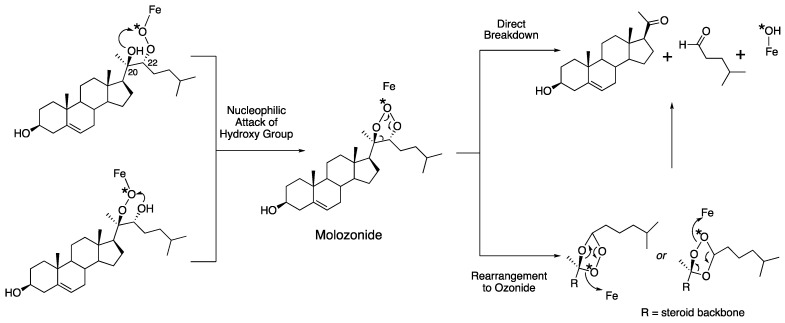
A molozonide mechanism, a derivative of the mechanism shown in Figure 4. The asterisk (*) indicates ^18^O used in labeling and its course in the reaction.

**Figure 6:**
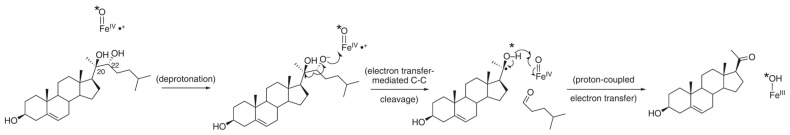
An alternate proposal for the C-C bond cleavage step based on theoretical calculations [65]. The asterisk (*) indicates ^18^O used in labeling and its course in the reaction.

X-ray crystal structures of P450 11A1 have been reported with both 22*R*-hydroxycholesterol (PDB 3MZS [59,60]) and 20*R*,22*R*-dihydroxycholesterol (PDB 3NA0) [60]. There has been general agreement that the enzyme uses a Compound I mechanism (Figure 2), based largely on the work of the Hoffman laboratory that demonstrated the catalytic competence of a Compound I entity generated by radiolysis [61,62]. Our work with ^18^O_2_ labeling [63] led to the conclusion that P450 11A1 Compound I acts as an electrophilic agent with one of the two hydroxyls of 20*R*,22*R*-dihydroxycholesterol (Figure 4), as opposed to abstracting a hydrogen atom from an alcohol [64]. An alternative mechanism involves a molozonide intermediate form with Compound I (Figure 5) [50]. Su et al. [65] have proposed an alternate scheme involving electron transfer from a deprotonated C22 oxygen atom to Compound I, based on theoretical calculations (Figure 6), which is still consistent with our own ^18^O labeling results [63]. The crystal structure of P450 11A1 with 20*R*,22*R*-dihydroxycholesterol has been reported (PDB: 3NA0) [60]. The distances between the C20-oxygen and the C22-oxygen of the ligand and to the heme iron are 3.3 and 3.6 Å, respectively. The closer distance of the C20 position could support the Compound I iron active species reacting at C20, as shown in Figure 4A, path b, to initiate the C20-C22 lyase reaction. However, the proposed mechanism may not be tenable in light of (i) the fact that all four 20,22-dihydroxycholesterol diastereomers are substrates for the last step [66,67,68,69] and (ii) two different rotamers are involved as intermediates (vide infra) [67], confounding through-space calculations.

**Figure 7:**
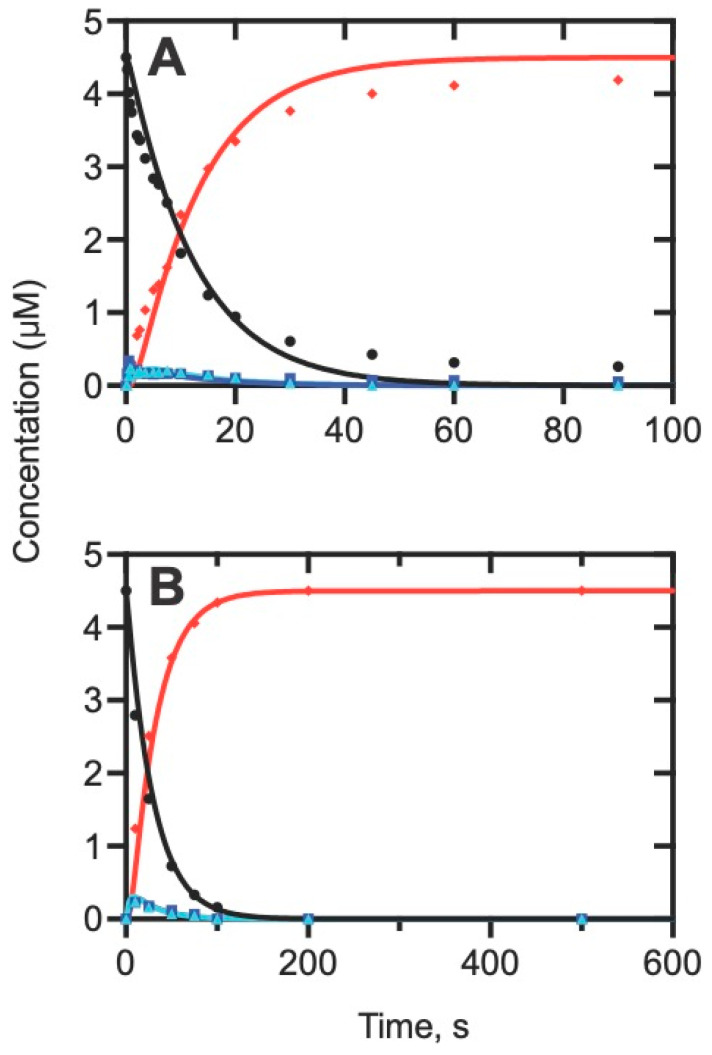
Time course of the reaction of 5 µM P450 11A1 with a limiting concentration of cholesterol [67]. Black (points and lines): cholesterol; light blue: 22*R*-hydroxycholesterol; dark blue: 20*R*,22*R*-dihydroxycholesterol; red: pregnenolone. (**A**,**B**) reflect different time scales.

**Figure 8:**
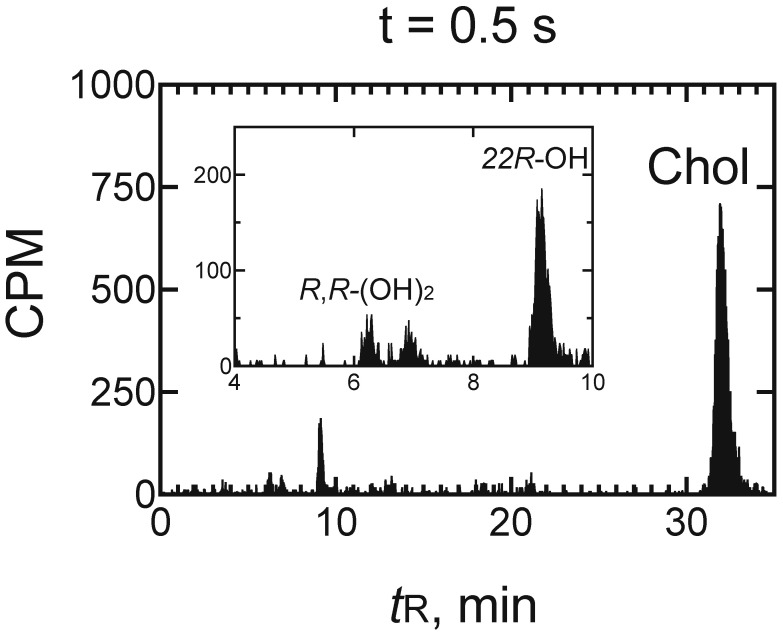
Radio-HPLC of the products of the reaction in Figure 7 (0.5 s). Note the presence of 22*R*-hydroxycholesterol (22*R*-OH) and two peaks in the region of 20*R*,22*R*-dihydroxycholesterol (*R*,*R*-(OH)_2_), one of which migrated at the position of standard 20*R*,22*R*-dihydroxycholesterol [67]. Chol: cholesterol; 22*R*-OH, 22*R*-hydroxycholesterol; *R*,*R*-(OH)_2_: 20*R*,22*R*-dihydroxycholesterol.

**Figure 9:**
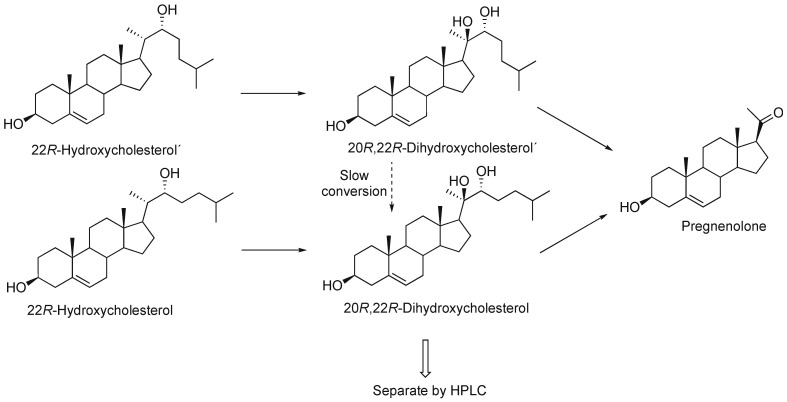
An explanation for the rotamers (conformers) in the experiment in Figure 8 [67].

The intermediates in the three-step reaction (Figure 3) are bound tightly, with low off-rates [67]. Thus, the reaction is *processive* as opposed to *distributive*, i.e., where the intermediate products have low affinity for the enzyme, dissociate, and have to be bound again before the next step (the oxidation step). As we will see later, such behavior (processivity) is also seen with P450 51A1 but not P450 19A1. P450 17A1 involves both processive and distributive aspects. Accordingly, the time course of a single turnover reaction shows only low levels of the 22*R*-hydroxy and the 20*R*,22*R*-dihydroxycholesterol intermediates (Figure 7) [67]. In the course of this work, two products were observed at short intervals in the dihydroxy product region on HPLC (Figure 8). Treatment of the (combined) products from this elution region with NaIO_4_ converted both products to pregnenolone, indicating that both were *vic*-diols, but in control reactions with only buffer, one was converted to the other, which migrated with standard synthetic 20*R*,22*R*-dihydroxycholesterol in HPLC [67]. We concluded that the two peaks are not diastereomers but rotamers, i.e., slowly converting conformers. Accordingly, these must result from the existence of two geometrically distinct complexes of 22*R*-hydroxycholesterol with P450 11A1 (Figure 9) [67]. The kinetics of formation and decay of these two conformers were very similar [67].

**Figure 10:**
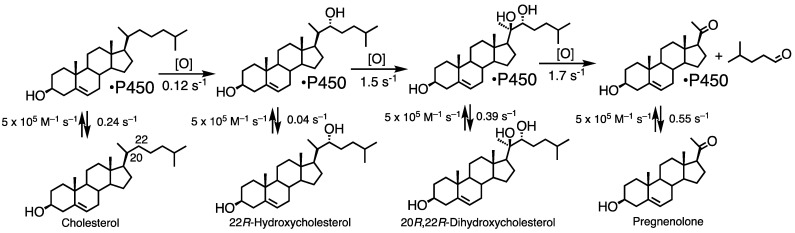
A scheme for the three-step oxidation of cholesterol by P450 11A1 with rate constants for steps derived from measurement of off-rates and global fitting to a single-turnover study (Figure 7) [67].

Kinetic modeling [70,71] yielded a scheme with the rate constants shown in Figure 10 [67]. No kinetic isotope effect was observed when the C-20 and C-22 hydrogens of cholesterol were substituted with deuterium [67], indicating that C–H bond breaking is not the rate-limiting step [72,73]. The reaction is characterized by a slow 22*R*-hydroxylation followed by two fast steps and the slow release of the intermediate sterols.

## 3. P450 11B2

**Figure 11:**
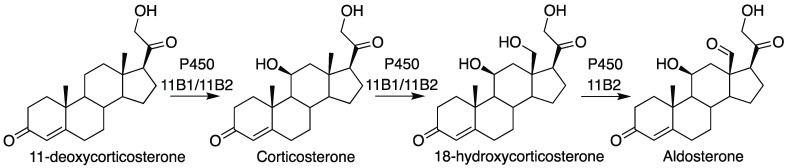
Conversion of 11-deoxycorticosterone into aldosterone by P450 11B2.

**Figure 12:**
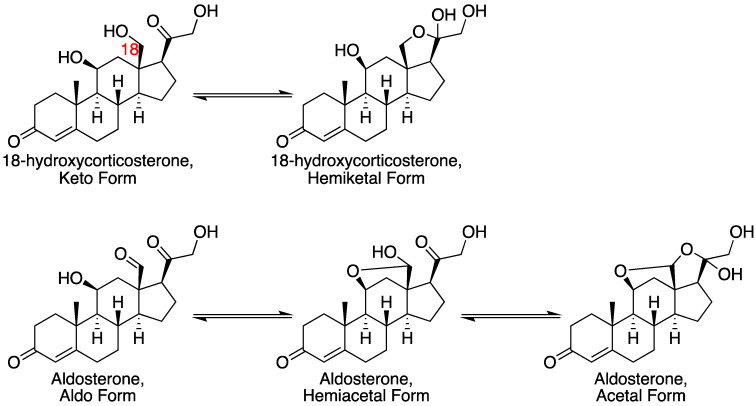
Conversion of 11-deoxycorticosterone into aldosterone by P450 11B2, with roles for acetal and ketal forms [78].The red 18 indicates the carbon number.

P450 11B2 catalyzes the three-step oxidation of 11-deoxycorticosterone to aldosterone (Figure 11) [74]. This process is important in the production of mineralocorticoids [75,76,77]. However, the enzyme is also a drug target in the case of several diseases, especially in the case of blocking aldosterone production [40,41,42]. The three-step process includes two hydroxylations (C-11 and C-18), followed by an oxidation of the C-18 alcohol to an aldehyde. A complication is that intermediates and the final product can exist in hemiacetal (lactol), acetal, and hemiketal forms (Figure 12) [78,79,80].

**Figure 13:**
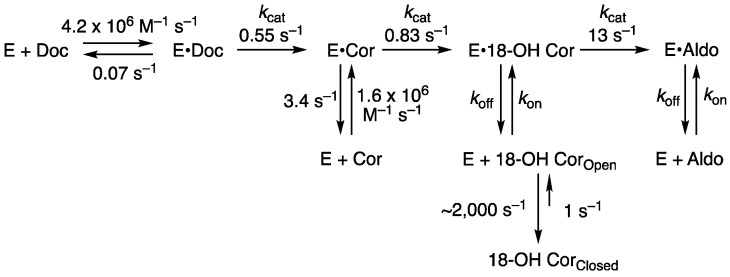
Kinetic model for the conversion of 11-deoxycorticosterone to aldosterone, with measured and fitted rate constants [78]. *k*_on_ and *k*_off_ rates for 18-hydroxycorticosterone (18-OH Cor) and aldosterone (Aldo) could not be measured due to the lack of spectral changes. See also Yalentin-Goyco et al. [81].

Single turnover experiments yielded an interesting pattern in that 18-hydroxycorticosterone was only converted to aldosterone, largely because of its propensity to cyclize to a form that cannot be readily oxidized (as demonstrated by NMR) [78]. Corticosterone appears to be a better substrate than 18-hydroxycorticosterone in that it can be oxidized to avoid ring closure. A model encompassing all the kinetic results is presented in Figure 13 [78].

## 4. P450 17A1

**Figure 14:**
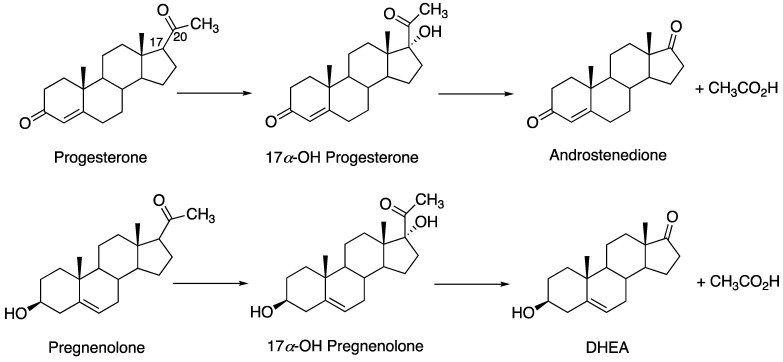
The major reactions catalyzed by P450 17A1.

P450 17A1 is a critical enzyme in the production of androgens. It has a number of very interesting features, some of which are still not well understood. The enzyme catalyzes two reactions: the 17α-hydroxylation of both progesterone and pregnenolone and the subsequent “lyase” reaction to generate the androgens androstenedione and dehydroepiandrosterone (DHEA), respectively (Figure 14). Prostate cancer is stimulated by androgens, and P450 17A1 is a drug target (e.g., abiraterone acetate (Zytiga^®^)) [36]. An inherent problem with the drugs is that most leads inhibit both the 17-hydroxylation and lyase steps. The first product, 17α-hydroxyprogesterone, is needed for the production of glucocorticoids (Figure 14). Therefore, patients with metastatic castration-resistant prostate cancer are treated with abiraterone acetate (the prodrug form of abiraterone) and prednisone, a glucocorticoid [82]. The “Holy Grail” in this case would be a drug that inhibits only the lyase step but not 17α-hydroxylation [39].

**Figure 15:**
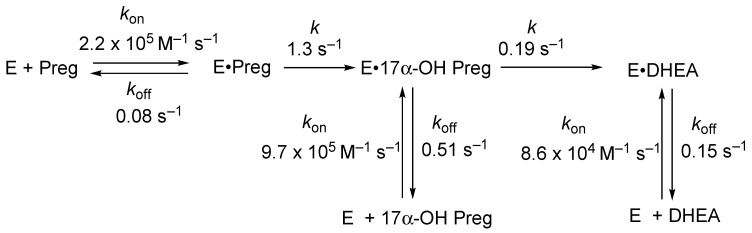
Conversion of pregnenolone into DHEA, with rate constants derived from direct measurements and the fitting of a single turnover reaction [83].

The overall reaction is partially processive (Figure 15) [83]. That is, only a fraction (~¼) of the DHEA or androstenedione is derived directly from pregnenolone or progesterone. Therefore, achieving selective inhibition of the second step is difficult in that not all of the intermediate (17α-hydroxysteroid) is dissociated.

**Figure 16:**
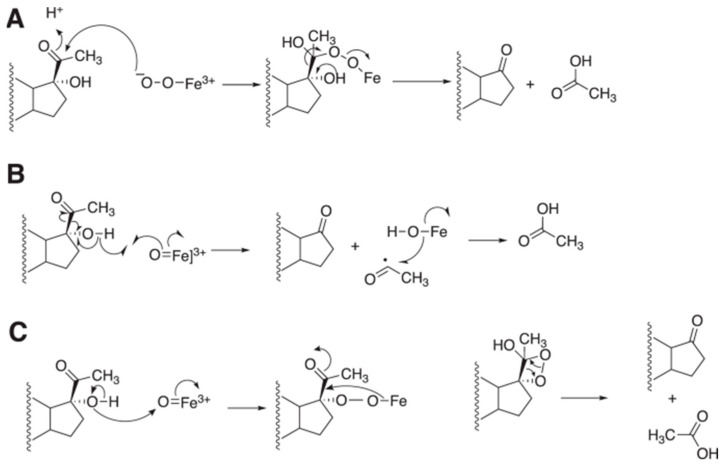
Alternate mechanisms for the P450 17A1 lyase reaction based on (**A**) Compound 0 and (**B**,**C**) Compound I [50].

**Figure 17:**
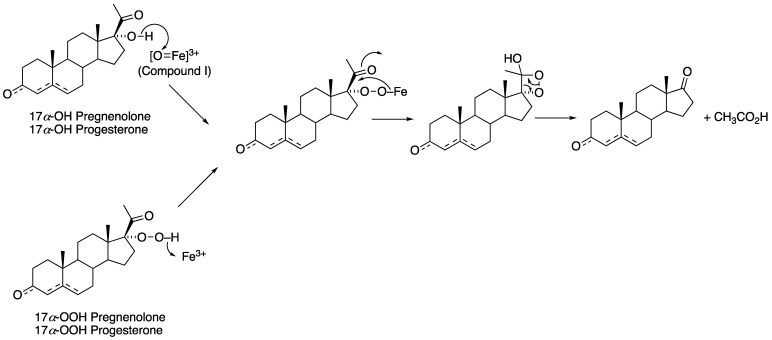
Reactions of 17α-OOH progesterone and 17α-OOH pregnenolone with ferric P450 17A1 to yield androstenedione and DHEA and relevance to normal mechanisms [86].

The mechanism of the 17α-hydroxylation reactions of P450 17A1 is generally accepted to be a straightforward Compound I hydroxylation (Figure 14). The question of whether the lyase reaction proceeds via a Compound I or a Compound 0 mechanism (Figure 2) has been controversial. ^18^O_2_ labeling experiments have been published, but the results (incorporation of ^18^O into acetate) are not unambiguous [84,85]. The lyase reaction can be supported by the use of the oxygen surrogate iodosylbenzene, which can only be interpreted in the context of a Compound I mechanism [85]. A proposed Compound I mechanism (Figure 16) is also consistent with the ability of progesterone 17α-hydroperoxides to generate the final products (androstenedione and DHEA) (Figure 17) [86]. Analysis of the crystal structures of P450 17A1 with its four different substrates can be achieved (P450 17A1 with 17α-hydroxyprogesterone, progesterone, 17α-hydroxypregnenolone, and pregnenolone) [87]. The distances between the C17-oxygen atom and the iron active site in the cases of 17α-hydroxyprogesterone and 17α-hydroxypregnenolone were measured to be 4.5 and 3.9 Å, respectively. Coupling the facts that 17α-hydroxypregnenolone is a better lyase substrate compared to 17α-hydroxyprogesterone for P450 17A1 (*k*_cat_ values for 17α-hydroxypregnenolone to DHEA and 17α-hydroxyprogesterone to AD were 0.35 min^−1^ and 0.019 min^−1^, respectively) [83], and the distance of the oxygen atom being closer to the iron in the active site for 17α-hydroxypregnenolone supports the mechanistic possibility of the C17-hydroxy of the lyase substrate attacking Compound I (Figure 17).

However, it is possible that the normal reaction does not necessarily occur this way. Swinney and Mak proposed a Baeyer–Villiger (Compound 0) mechanism based on the presence of 17-acetoxytestosterone as a minor product in a progesterone reaction with hog liver microsomes [88]. However, we were unable to find this product (or testosterone) in a purified human P450 17A1 reaction [85]. Mak et al. added O_2_ to ferrous P450 17A1 and then an extra electron (from ^60^Co radiation) at low temperature (and in a high glycerol concentration)—resonance Raman spectra were reported, and the spectra changed upon heating [89]. This complex was concluded to be Compound 0, but no product was reported (i.e., catalytic competence was not demonstrated).

Other approaches have also been used to study the mechanism of the C-C bond lyase reaction (Figure 16). Khatri et al. [90] noted that the lyase reactions of the enzyme were attenuated much more than the 17α-hydroxylation reactions by introducing a T306A mutation, which they interpreted as evidence that Thr-306 is involved in a proton transfer step in the (Compound I) 17α-hydroxylation but is not so necessary in the lyase because it may involve a Compound 0 intermediate. Another approach is the artificial generation of putative intermediates and characterizing them by spectroscopy. This has been done with P450 17A1, and the spectra have been interpreted in the context of Compound 0 [89,91], although a caveat is that the formation of the product was not addressed (i.e., catalytic competence).

Both Swinney and Mak [92] and Gregory et al. [93] have used arguments about solvent kinetic isotope effects (KIE) (*k*_H2O_/*k*_D2O_) to argue for a Compound 0 reaction, although the conclusions are in opposite directions (i.e., both a positive and an inverse solvent KIE have been proposed to support a Compound 0 mechanism [92,93,94]). Both arguments can be considered moot in light of the general criticisms raised earlier by others about solvent KIEs, including Jencks [95,96]. Placing a protein in D_2_O changes hundreds of protons (protium) with deuterium, and the effects of global deuteration on structure and hydrogen bonding are not really interpretable [72,95,96,97,98] (in 1959, it was already established that the *T*_m_ of RNase was changed by 4 °C in D_2_O [99]). In conclusion, none of the approaches used to date can be used to make a definite conclusion about the normal reaction mechanism, in light of the ambiguity of the ^18^O_2_ approach with α-ketol (α-hydroxyketone) substrates.

**Figure 18:**
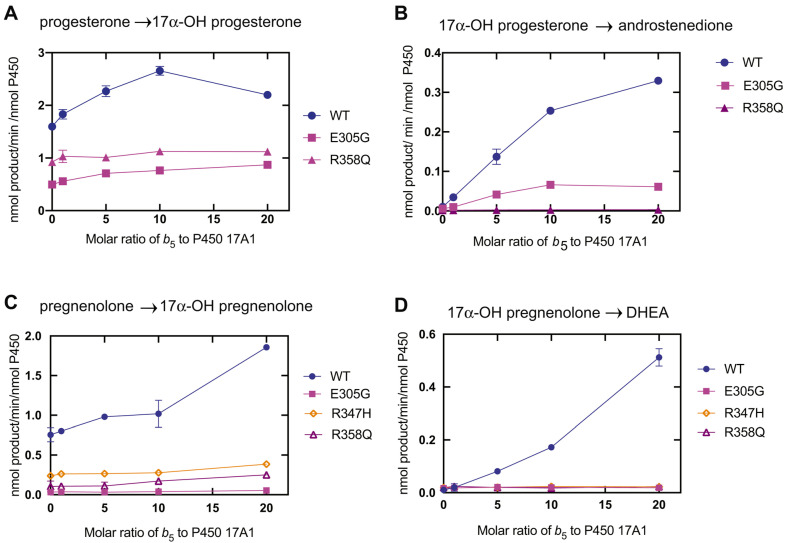
Effects of *b*_5_ on the 17α-hydroxylation and 17α,20-lyase steps of P450 17A1 and site-directed mutants devoid of lyase activity [102].

The 17-hydroxylation reactions are only slightly stimulated by cytochrome *b*_5_ (*b*_5_), but the lyase reaction is almost completely dependent on this accessory protein [100,101,102,103,104,105] (Figure 18). The mechanism for the *b*_5_ stimulation is generally considered to be an allosteric one, which is the case with many of the other mammalian P450s that show *b*_5_ stimulation [106,107]. Although *b*_5_ has been shown to be capable of transferring an electron to the Fe^3+^O_2_ P450 17A1 complex [108], numerous other studies have shown that the heme moiety of *b*_5_ is not necessary for stimulation [109,110], even in mammalian cells [111].

**Figure 19:**
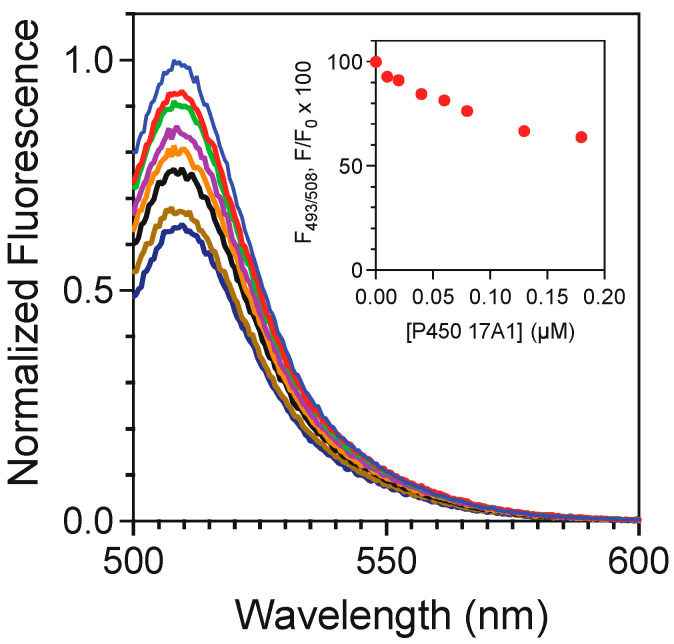
Binding of *b*_5_ to P450 17A1 as demonstrated by titration of AlexaFluor 488-labeled *b*_5_ with P450 17A1 [102,112]. The individual traces correspond to increasing P450 17A1, and the concentrations are indicated in the inset.

**Figure 20:**
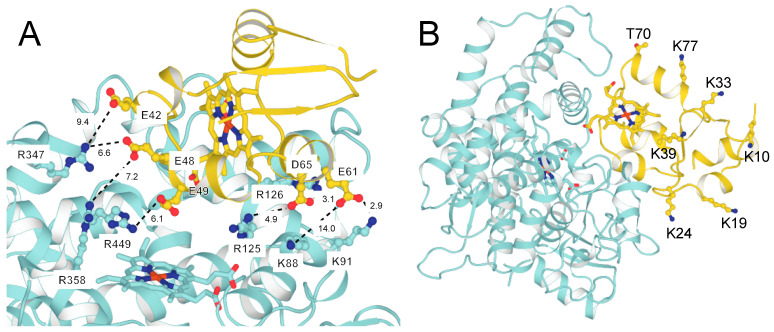
(**A**) Model of interaction of human P450 17A1 (blue) and *b*_5_ (yellow) (developed with AlphaFold-Multimer and Rosetta programs) [112]. (**B**) The same complex as in A but with the entire P450 section and showing potential sites of interaction on the *b*_5_.

**Figure 21:**
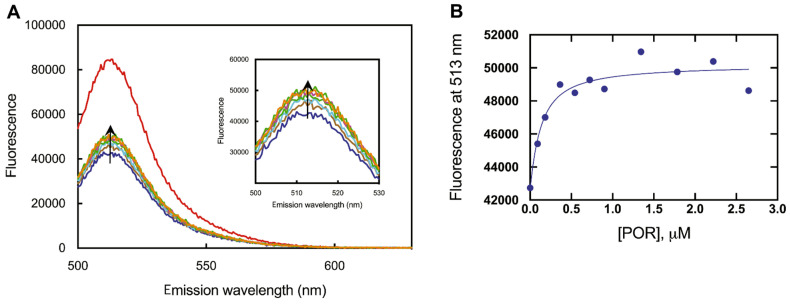
Titration of a P450 17A1–AlexaFluor 488 complex with POR [102]. (**A**) Titration, with expansion in the inset. The arrows show the direction of the changes after adding increasing conentrations of POR. (**B**) Plot of the F_513_ data from (**A**).

**Figure 22:**
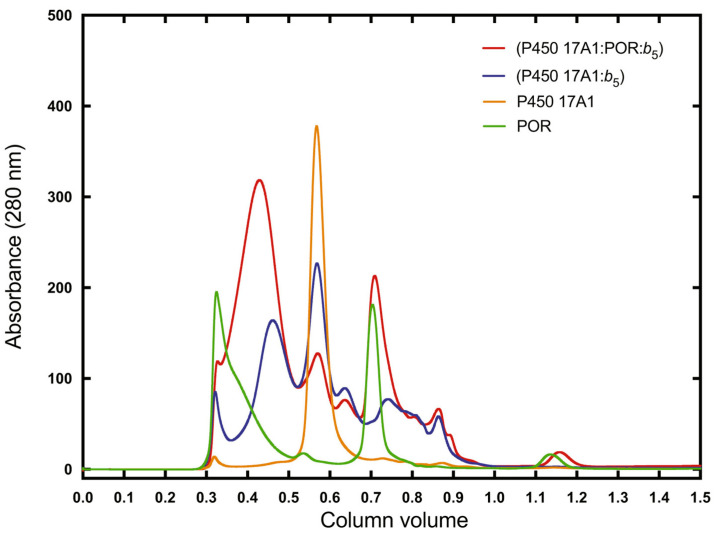
Demonstration of a ternary P450 17A1–POR–*b*_5_ complex (red trace) by gel filtration [102].

**Figure 23:**
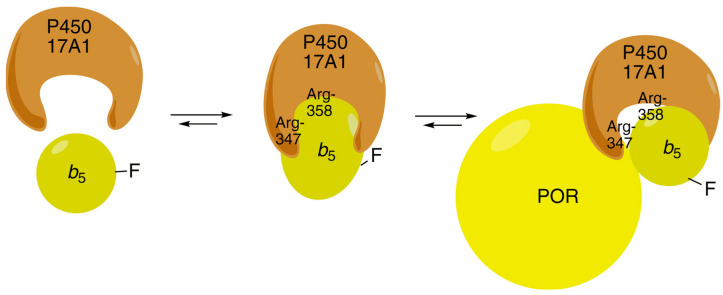
Scheme to demonstrate the changes in AlexaFluor 488 fluorescence observed in the context of a ternary complex (Figure 19 and Figure 20) [102]. Arg-347 and Arg-358 are on P450 17A1 (Figure 20).

*b*_5_ binds tightly to P450 17A1, as shown in titrations with AlexaFluor 488-labeled *b*_5_ (Figure 19) [102,112]. The affinity is in the range of *K*_d_ from 120 to 380 nM, as judged by the fluorescence polarization assay using modified *b*_5_ variants [112]. A model has been developed for the docking of *b*_5_ and P450 17A1, consistent with reported chemical cross-linking results [113] (Figure 20) [112]. Although it has been proposed that *b*_5_ and NADPH-P450 reductase (POR) both bind to the same site on P450 17A1, based on NMR measurements [114,115], this scenario would require switching of redox partners in every reaction cycle, at a stage in which unstable high-valent intermediates (e.g., Fe^3+^O_2_¯) exist. When a complex of P450 17A1 and Alexa 488·*b*_5_ was titrated with POR, the fluorescence was only partially restored (Figure 21) [102,112], providing evidence for a ternary P450 17A1–POR–*b*_5_ complex. The existence of such a complex could also be demonstrated using gel filtration (Figure 22) [102]. The results can be explained in a model with a ternary complex in which the addition of POR only perturbs the position of the *b*_5_ (Figure 23).

Although cross-linking data are available and support a model for the interaction of P450 17A1 and *b*_5_ (Figure 20), a more complete understanding of the effect of *b*_5_ on P450 17A1 and the basis of the lyase activity will probably require an X-ray crystal structure of a binary complex.

## 5. P450 19A1

P450 19A1 is the steroid aromatase, which converts androgens to estrogens [116,117,118,119,120,121,122,123]. There is a single gene, but differential splicing leads to the production of the enzyme from individual mRNA species in different tissues, e.g., adipose tissue, brain, ovary, and placenta [124,125,126,127]. The regulation of the gene is complex [128,129,130,131,132,133,134,135,136,137,138,139]. Deficiencies are serious in that androgen synthesis is attenuated [29,140,141,142,143,144,145,146,147]. However, a number of female endocrine cancers are stimulated by estrogens, and the inhibition of the enzyme is now an established approach to therapeutic interventions against these cancers [42,148,149,150,151,152,153,154,155,156,157,158,159,160,161,162,163,164]. Although most attention has been given to this enzyme as a “female” P450, it is present in males and important in brain development [120,165] and is even present in the penis [166,167].

**Figure 24:**
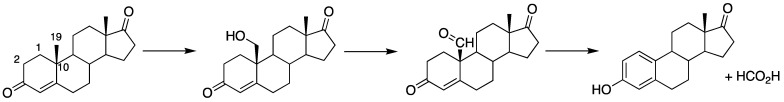
Three-step oxidation of androstenedione to estrone by P450 19A1. A similar reaction is involved in the oxidation of testosterone to 17β-estradiol.

**Figure 25:**
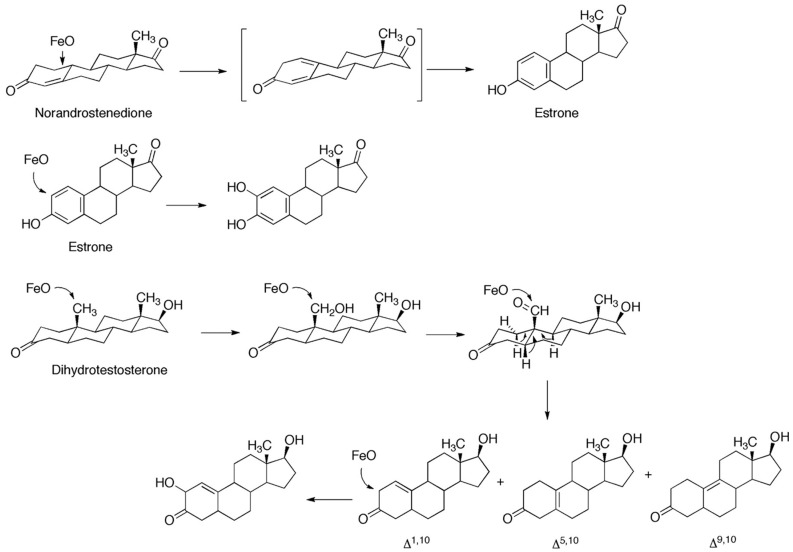
Some other reactions catalyzed by P450 19A1 [168,169].

The major three-step reactions are the two shown in Figure 24, with the substrates androstenedione and testosterone. Other reactions include the 2-hydroxylation of estradiol [168], the oxidation of 4,5-dihydrotestosterone to three 3-keto unsaturated steroids (Figure 25), and the oxidation of 19-oxo steroids to carboxylic acids [169,170].

**Figure 26:**
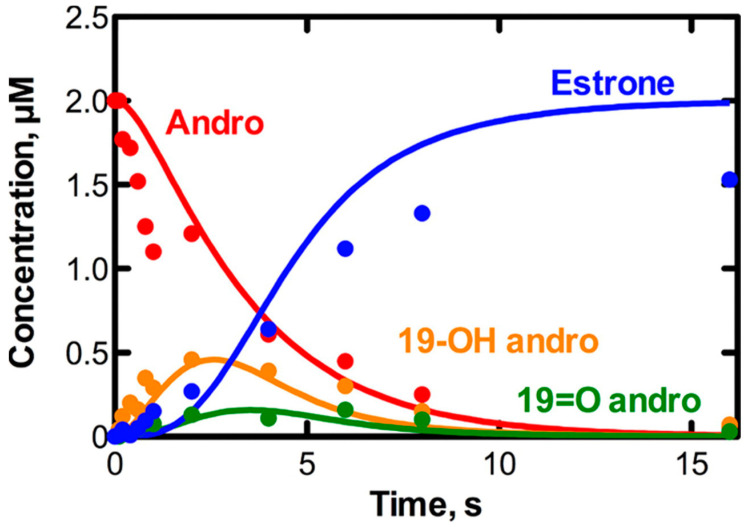
Single turnover results for the conversion of androstenedione to estrone by human P450 19A1 [171]. Andro, androstenedione; 19-OH andro, 19-hydroxyandrostenedione; 19=O andro, 19-oxo androstenedione.

**Figure 27:**
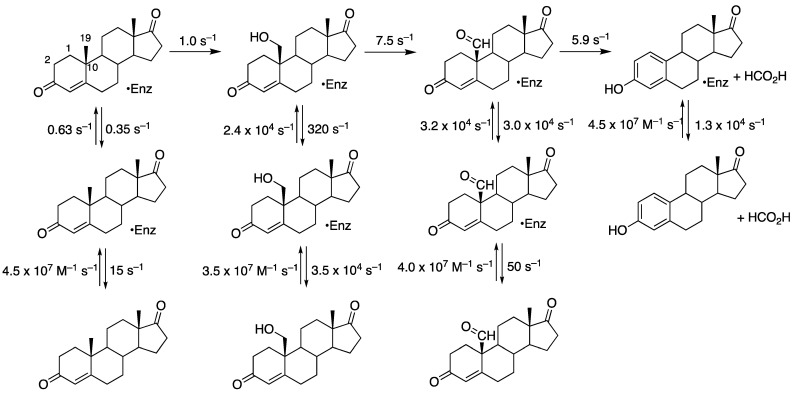
A kinetic scheme for the three-step oxidation of androstenedione to estrone based on direct assays and the fitting of a single-turnover reaction [171].

In contrast to the situation with P450s 11A1 (Figure 10) and P450 51A1 (vide infra), the three-step sequence with P450 19A1 is a kinetically distributive one (Figure 26 and Figure 27) [171], with the intermediate products readily dissociating from the enzyme and rebinding.

**Figure 28:**
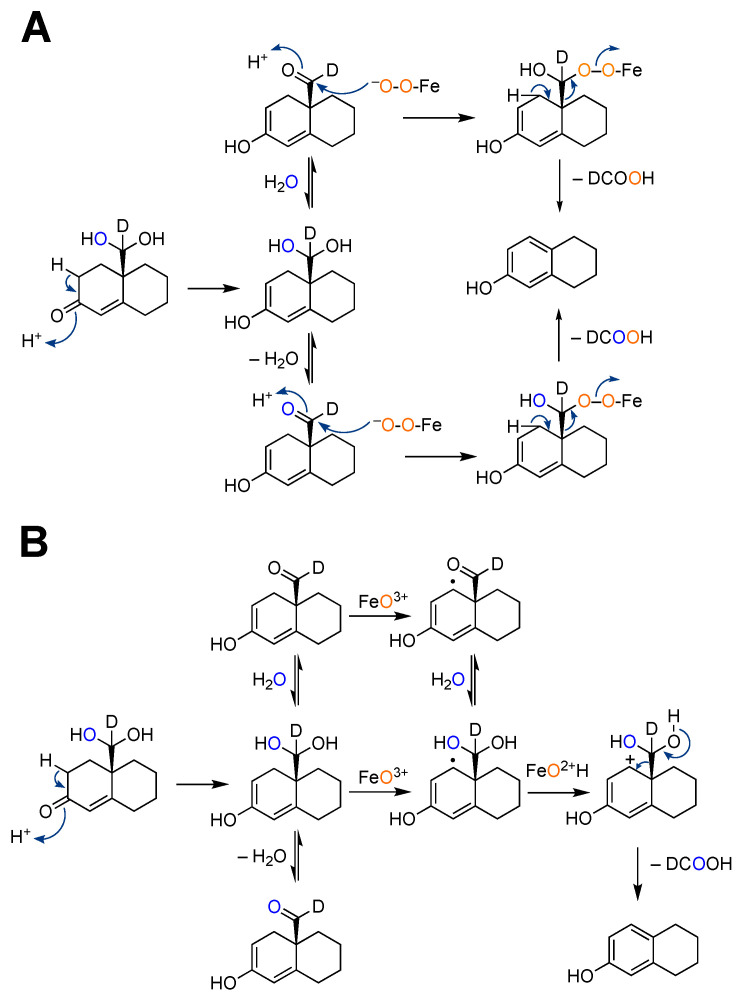
Alternate mechanisms proposed for the third oxidation step of P450 19A1 [183]. (**A**) Compound 0; (**B**) Compound I. Only steroid A and B rings are shown.

The mechanism of the third step, in which the 10-formyl (19-oxo) group is released as formic acid, has been the object of many studies since the 1970s. A number of approaches have been applied, including biomimetic models [172,173,174,175], computational modeling [176,177,178,179,180], spectroscopy of proposed intermediates [181,182], isotopic labeling and analysis of products [183,184,185], solvent kinetic isotope effects [186], and site-directed mutagenesis [187,188]. Both Compound 0 and Compound I mechanisms have been proposed (Figure 28).

**Figure 29:**
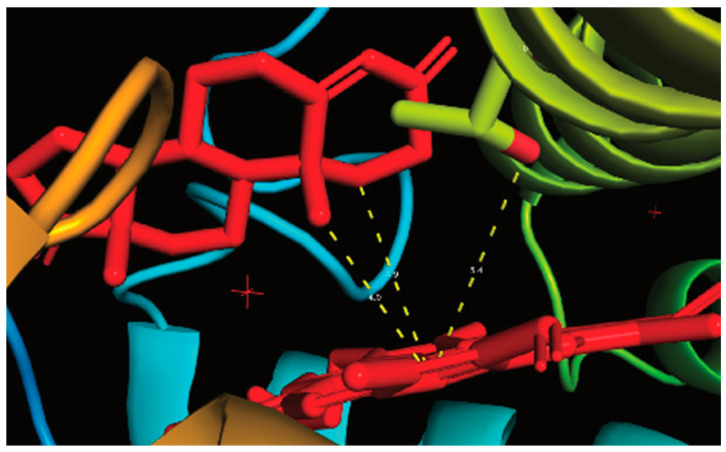
Interior of the structure of human P450 19A1 with bound androstenedione (X-ray crystal structure from Protein Data Bank 3EQM). The dashed lines indicate the distance from the heme iron atom to (i) the C19 atom (of the substrate) (4.0 Å), (ii) the C1 carbon (4.9 Å), and (iii) the Thr-310 oxygen atom (5.4 Å). (The + symbols are reference markers.)

An X-ray structure of human P450 19A1 with bound androstenedione (Figure 29) indicates that both the C1 and C19 carbon atoms are in close proximity to the iron atom of the heme. Thus, this structural information does not distinguish between the potential catalytic mechanisms (Figure 28).

**Figure 30:**
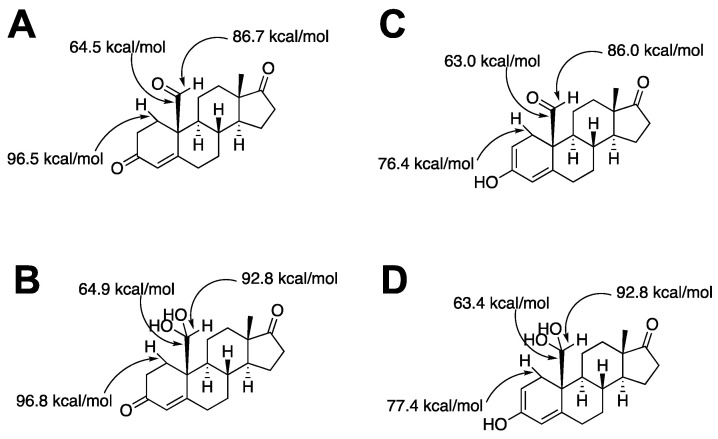
Calculations of C-C and C-H bond dissociation energies for potential forms of 19-oxoandrostenedione bound to P450 19A1. Bond energies were calculated using ALFABET (National Renewable Energy Laboratory, bde.ml.nrel.gov) (accessed on 17 August 2024) [189,190]. The tautomers are shown for the aldehyde (**A**,**C**) and the *gem*-diol (**B**,**D**). A and B are the keto forms, and C and D are the enol forms. See Figure 28.

Bond energy calculations are also of potential interest (Figure 30). The free energy for breaking the C10-C19 bond is similar in all tautomers. When the aldehyde group is hydrated, the bond energy rises. However, if the androgen is in the enol form, the C1-H bond energy is decreased considerably (Figure 30C,D). Even with the crystal structure of the P450 19A1–androgen complexes, though, we do not know which tautomer is favored.

One approach to distinguishing the roles of Compounds I and 0 in P450 reactions is with a single-oxygen donor oxygen surrogate (e.g., iodosylbenzene) that can support the reaction [191,192,193]. These experiments must be interpreted carefully, in that iodosylbenzene destroys P450 quickly. Neither iodosylbenzene, periodate, nor cumene hydroperoxide was able to catalyze the overall P450 19A1 reaction [194,195], although some conversion of 19-oxo androstenedione to estrone was reported with *m*-chloroperbenzoic acid [188].

The Sligar laboratory has interpreted their results with P450 19A1 in favor of a Compound I role in the activity of P450 19A1 [181,186], although neither the solvent KIE nor the Raman spectroscopy studies can be considered unambiguous in the elucidation of catalytic mechanisms (vide supra). Zhang et al. [188] prepared what was considered to be P450 19A1 Compound I using *m*-chloroperoxybenzoic acid (as in the case of the work of Rittle and Green with P450 119 [196]) and demonstrated the aromatization of 19-oxo androstenedione (to estrone) with it, establishing some catalytic competence, at least under these conditions.

One of the most definitive approaches is isotopic labeling, in that the origin of the oxygen in the formic acid provides information about the mechanism within the context of the normal enzyme reaction (Figure 28). The experiments are technically difficult in that (i) the need for anaerobicity prior to the introduction of an ^18^O_2_ atmosphere is critical and (ii) the contribution of endogenous (^16^O) formic acid is very problematic—the only realistic means of overcoming this is with the use of a substrate with deuterium substitution on the aldehyde group to shift the mass of the derivatized formic acid. Further, any non-enzymatic degradation of the substrate and release of DCO_2_H will give nebulous results. The approach was developed by Akhtar and associates [183,184,197,198,199,200] and modified in our own group [85,201,202].

In light of the significance of previous ^18^O incorporation results [183,184,203] and their dominance in the dogma regarding the mechanism [45,64,197,198], we repeated the ^18^O study using purified recombinant human P450 19A1 and introduced two other major technical improvements—the use of (i) a new diazo-based derivatizing reagent that allowed for high-sensitivity analysis of a formic acid ester and (ii) high-resolution mass spectrometry (HRMS). However, this derivative is still problematic in mass spectrometry due to the presence of natural abundance ^13^C and confusion between DCO_2_R and H^13^CO_2_R products, which have the same unit *m*/*z* values. HRMS (at a resolution > 60,000) can readily discern these species, however [170]. The appropriate controls ruled out exchange of oxygen between formic acid and water, and the reported total incorporation of ^18^O into the side product androstenedione 10-carboxylic acid rules out the possibility of no ^18^O_2_ being present in the gas atmosphere in any particular experiment [170].

In the course of our studies with the model secosteroid 3-oxodecalin-4-ene-carboxyaldehyde (ODEC) [204], we noted the acid instability of ODEC. We improved our analysis of formic acid by (i) lowering the acid concentration used during extraction (only needing to protonate the formic acid), (ii) changing the extraction solvent from CH_2_Cl_2_ to *tert*-butyl methyl ether, (iii) omitting the MgSO_4_ drying step for the extracted formic acid solution, and (iv) adding 10% CH_3_OH (*v*/v) to the diazotization reaction [202]. Collectively, these modifications led to a three-order-of-magnitude increase in sensitivity. In addition, we included a P450 17A1-progesterone reaction yielding ^18^O-labeled 17α-OH progesterone as an internal standard to correct for any leakage into the ^18^O_2_ atmosphere. An important control was the addition of minus-NADPH control incubations, which had not been included earlier [170,183,184,185] and provides a check on the extent of non-enzymatically generated deuterated formic acid, which would be interpreted as a lack of ^18^O_2_ labeling of formic acid.

**Figure 31:**
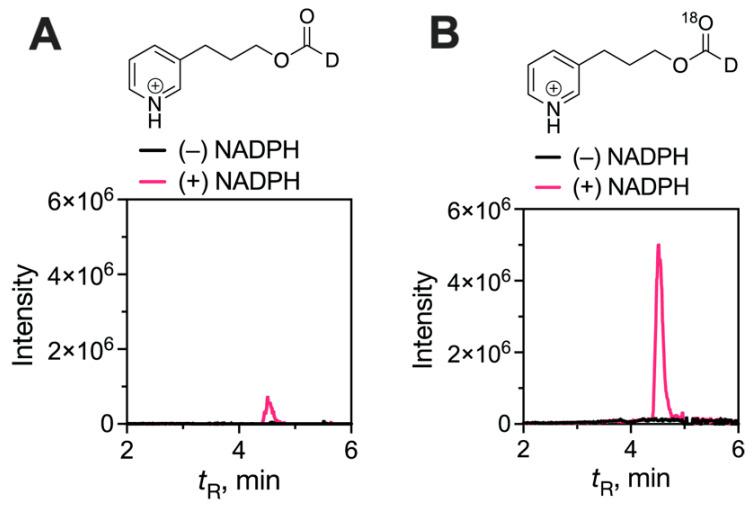
Results of the ^18^O_2_ labeling study and formic acid analysis to distinguish between Compound 0 and Compound I mechanisms for human P450 19A1. The Compound 0 mechanism should yield one ^18^O atom in formic acid, and the Compound I will not yield any ^18^O in formic acid (Figure 26) [170,195]. (**A**) ^16^O channel data; (**B**) ^18^O channel data.

**Figure 32:**
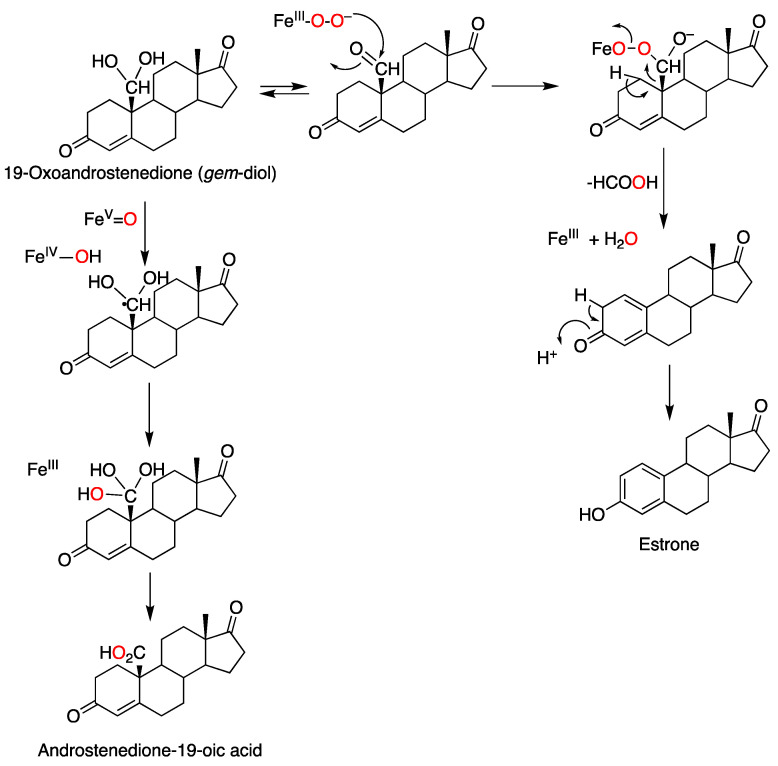
Conclusions about the third step of P450 19A1 based on ^18^O labeling (Figure 26 and Figure 27) [195]. Alternatively, the formation of the 19-oic acid could be initiated via hydrogen atom abstraction from the aldehyde by the Compound I intermediate, followed by oxygen rebound, which would be more consistent with the complete ^18^O incorporation results [170]. The red color is used to track the course of the oxygen atoms in the reaction in the schemes.

The results now show nearly complete incorporation of one atom of ^18^O from ^18^O_2_ (91%) [195]. The results were confirmed with the incorporation of only one, not two, ^18^O oxygen atoms into formic acid when ^18^O-labeled 19-oxo androstenedione was incubated in H_2_^18^O under air, consistent with the ^18^O_2_ labeling pattern (Figure 31). Accordingly, we conclude that the ^18^O labeling patterns provide evidence for a very dominant Compound 0 (FeO_2_¯) mechanism, in contrast to our earlier conclusions [170] (Figure 31 and Figure 32).

We are further characterizing the chemistry of the general instability of allyl formyl derivatives of ∆4-seco steroids (e.g., 19-oxo androstenedione and ODEC). As alluded to by Houghton et al. [205], we found that androstenedione 10-carboxylic acid readily undergoes degradation, presumably with the loss of CO_2_ [195], to yield 19-norandrostenedione. 19-Norandrogens are physiological products, apparently without known function [205,206,207].

## 6. P450 51A1

**Figure 33:**
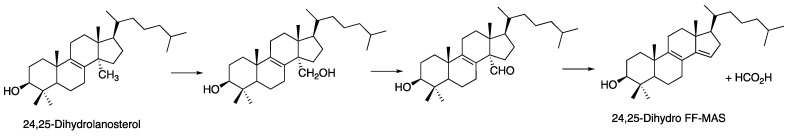
Overall conversion of (24,25-dihydro)lanosterol to 24,25-dihydro-4.4-dimethyl-5α-cholesta-8,14,24-trien-3β-ol (24,25-dihydro FF-MAS) by mammalian P450 51A1 and other P450 51 enzymes.

This is the only P450 involved in the synthesis of cholesterol. It cleaves the 14α-methyl group in a three-step reaction (Figure 33). Orthologues of the enzyme in yeast, fungi, and other parasites [208] are involved in the synthesis of critical membrane sterols (e.g., ergosterol, necessary for membranes) and are important drug targets [209,210,211,212,213,214,215,216,217].

**Figure 34:**
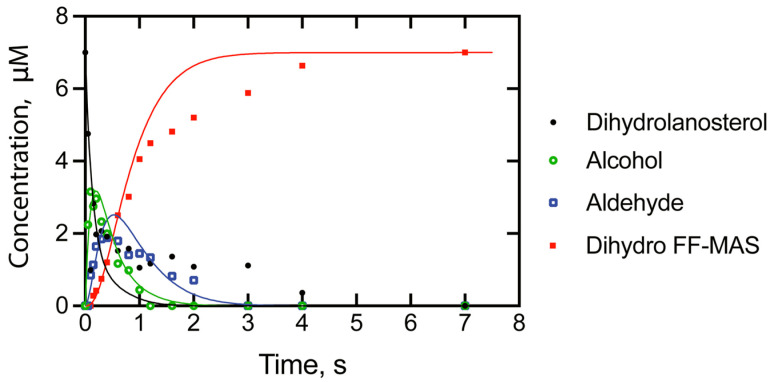
Time course of conversion of 4.5 µM [3-^3^H]-24,25-dihydrolanosterol by 5 µM human P450 51A1, fit to a kinetic model [218]. The colors of the lines correspond to the fits for the substrate and different products (see legend at right of graph).

**Figure 35:**
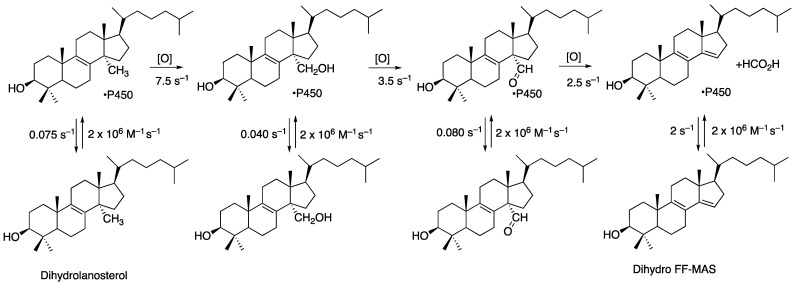
Scheme for three-step oxidation of dihydrolanosterol to dihydro FF-MAS with rate constants included from direct measurements or fitting from the time course data of Figure 30 [218].

The overall sequence is highly processive, but not as much as in the case of P450 11A1. The processivity is apparent in a single-turnover study (Figure 34) [218]. Fitting of the kinetics yields a scheme with individual rate constants (Figure 35). In the initial step, C-H bond breaking is not rate-limiting, in that no deuterium KIE was observed with cholesterol in which the oxidized carbon atoms were substituted with deuterium [218].

**Figure 36:**
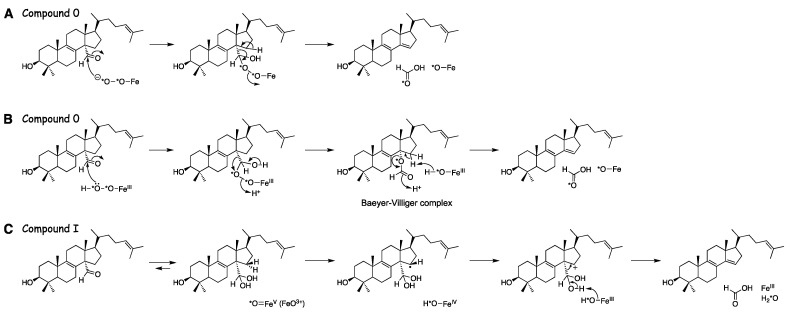
Three proposed mechanisms for P450 51 family enzymes. (**A**) Compound 0. (**B**) Compound 0 with a Baeyer–Villiger rearrangement. (**C**) Compound I. Tracking of the individual oxygen atoms into products, especially formic acid, is indicated with asterisks.

As in the cases of several other P450s, conflicting conclusions have been advanced about the roles of Compound I and Compound 0 in the final deformylation step [219,220,221,222,223]. The major possibilities are shown in Figure 36, with the oxygen in O_2_ labeled. Shyadehi et al. [224] reported an ^18^O_2_ experiment with *Candida albicans* P450 51 in which 65% ^18^O was recovered in the formic acid, but the recovery of deuterated formic acid was low and the unnatural ∆7 isomer of 24,25-dihydrolanosterol had been used (not the natural ∆8).

**Figure 37:**
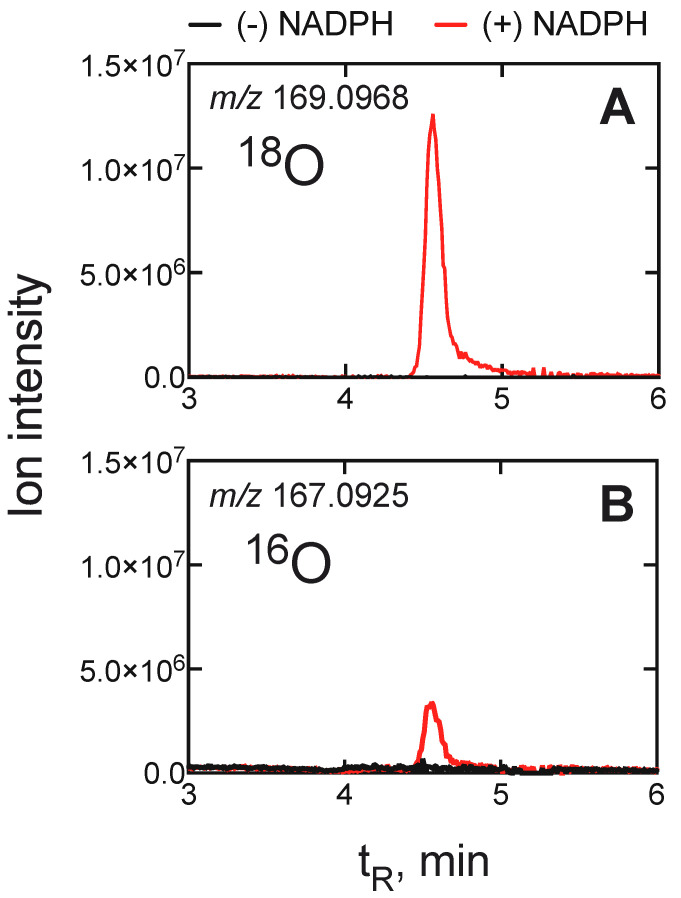
HPLC-HRMS analysis of the ester derived from *d*_1_-formic acid generated from 14-CDO dihydrolanosterol by human P450 51A1 [202]. (**A**) Ion trace channel for *m*/*z* 169.0968. (**B**) Ion trace channel for *m*/*z* 167.0925.

**Figure 38:**
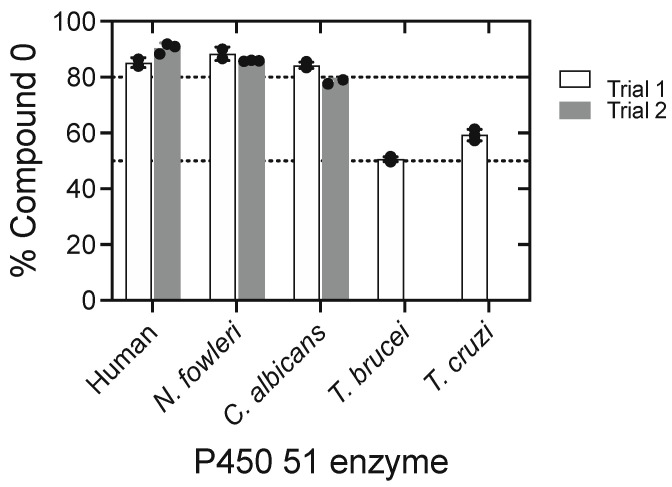
Extent of ^18^O_2_ incorporation into formic acid by several P450 family 51 enzymes [202]. The stippled lines are set at the 50 and 80% levels. The black dots are the results of individual experiments, plus the mean ± standard deviation calculated for each set.

We synthesized 14α-formyl-deuterated (24,25-dihydro) lanosterol (also called lanostenol) and recovered formic acid with 0.86 atoms of ^18^O after normalization (Figure 37), indicating that a Compound 0 mechanism was dominant [202]. The 86% result could be interpreted as only being a Compound 0 reaction, but other work in this laboratory with P450 2B4 and some aldehyde deformylation reactions yielded > 95% ^18^O incorporation under the same conditions [201], and the statistical variance was small. Experiments with the P450 51 enzymes from the yeast *Candida albicans* and a pathogenic amoeba, *Naegleria fowleri*, also yielded high incorporation of ^18^O, indicative of Compound 0, but two trypanosomal P450 51 enzymes (*Trypanosoma cruzi* and *T. brucei*) yielded ~50% (Figure 38). These experiments suggested a partial role for a Compound I mechanism. This conclusion was verified in assays with H_2_^18^O, in which the oxygen in the formyl group had been exchanged with ^18^O. In this experiment, the formic acid contains one ^18^O in the Compound 0 mechanism but two ^18^O atoms in the Compound I mechanism (Figure 36) [202].

**Figure 39:**
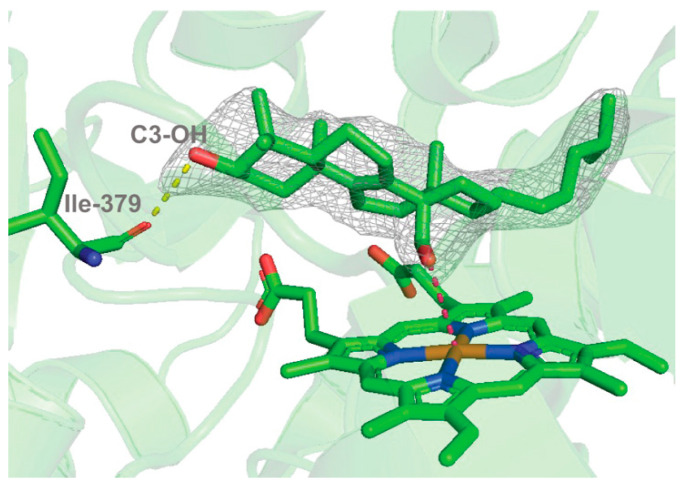
Substrate-bound human P450 51A1. Binding mode of the 14α-aldehyde reaction intermediate inside the enzyme active site (PDB 8SS0, 2.25 Å.) [202]. The 2*F*_o_-*F*_c_ electron density map within 1.6 Å of the sterol atoms is shown as a gray mesh and contoured at 1.5σ. The H-bond between the C3-OH of the sterol molecule and the main chain oxygen of Ile-379 is depicted as yellow dashes. The distance between the aldehyde oxygen and the heme iron is 3.5 Å (pink dashes).

An X-ray crystal structure of P450 51A1 with the 14α-formyl lanosterol derivative (Figure 39) showed the aldehyde form of dihydrolanosterol, with the oxygen of the formyl group pointed towards the iron atom, only 3.5 Å away.

**Figure 40:**
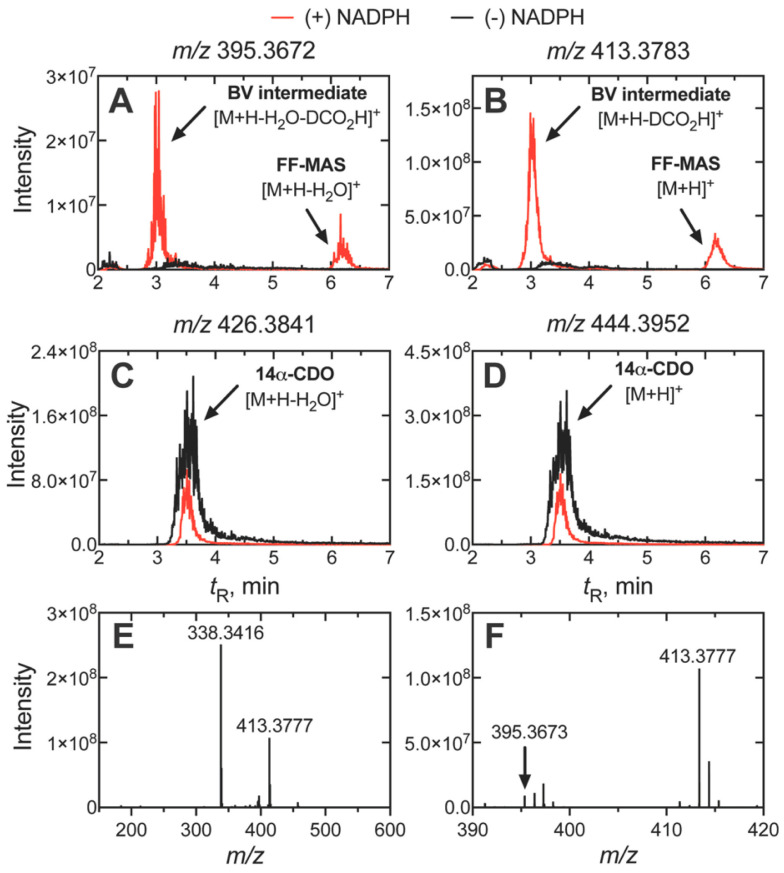
HPLC-HRMS evidence for a Baeyer–Villiger (BV) intermediate in the 14α-deformylation reaction catalyzed by human P450 51A1 [202]. (**A**) traces of products with a loss of 18 a.m.u. (H_2_O) and DCO_2_H for the BV complex; (**B**) traces of BV intermediate (minus DCO_2_H) and FFMAS; (**C**,**D**) traces of substrate with and without a loss of H_2_O; (**E**,**F**) spectra of BV intermediate (note different *m*/*z* scales). See also Fischer et al. [219].

One variation of the Compound 0 mechanism is a Baeyer–Villiger rearrangement (Figure 34), which is common in flavin 4a-hydroperoxide-based reactions [225,226,227,228,229,230]. Evidence for such an intermediate had been reported in experiments with rat liver microsomes and radiolabeled lanosterol by Fischer et al. [219]. We were able to identify what appears to be this Baeyer–Villiger intermediate in the oxidation of dihydrolanosterol by purified human P450 51A1 using both reversed-phase LC-HRMS (Figure 40) and normal phase LC-MS, employing similar chromatographic systems as Fischer et al. [202,219].

We conclude that P450 51 enzymes use multiple mechanisms to catalyze the deformylation in the last oxidation step (Figure 36). A Compound 0 mechanism is used ~ 85% of the time (for the human enzyme and those of *C. albicans* and *N. fowleri*), and a Compound I mechanism is used ~15% of the time. A Baeyer–Villiger rearrangement also occurs, but the extent to which this reaction occurs—as opposed to a “direct” Compound 0 mechanism—is presently unknown (Figure 36B). Fischer et al. [219] isolated the Baeyer–Villiger ester but found it very sensitive to acid-catalyzed decomposition, and we would probably not have detected this in our kinetic analyses (Figure 34 and Figure 35).

One possibility is that the Compound I mechanism is more favorable in enzymes that have slow rates of 14-deformylation (of dihydrolanosterol) because the Compound 0 intermediate is not intercepted so quickly by the formyl substrate. Perhaps similar isotopic studies with the natural sterol substrates (not lanosterol or dihydrolanosterol) for the trypanosomal P450s (i.e., obtusifoliol [222,231,232]) would yield different results. Alternatively, the explanation for the differences could be the rate of protonation of Complex 0 in the different P450s.

## 7. Summary and Conclusions

Five major multi-step steroid oxidations have been studied in this laboratory, those involving human P450s 11A1, 11B2, 17A1, 19A1, and 51A1. Our focus has been on two issues: (i) the processivity of the reaction steps and (ii) the roles of different iron-oxygen complexes in catalysis. Although one might expect to find a commonality among the P450s we have examined, what we have seen is diversity. Perhaps that should not be a surprise, in that P450s catalyze such diverse reactions in nature [43,233,234,235].

With regard to kinetics, P450s 11A1 and 51A1 are highly processive [67,218]. P450 17A1 is partially processive [83], which makes some biological sense in that the intermediates are used for other physiological functions, i.e., the synthesis of glucocorticoids (Figure 14). However, P450 19A1 has distributive kinetics [171], but it is not clear that the intermediate products have uses (although androstenedione 19-carboxylic acid is a known biological entity without an assigned function [236]). The situation with P450 11B2 is complex in that the involved chemistry locks an intermediate and makes it difficult to oxidize, both in vitro [78] and in vivo [79,237,238]. If there is a biological reason for this, it might be to regulate aldosterone production.

The involvement of different high-valent iron–oxygen species in catalysis is still not without controversy [50,51], but some of the reactions now have explanations. The P450 11B2 oxidations are chemically straightforward and probably all involve Compound I. All three of the P450 11A1 reactions are attributed to Compound I [61,62], although the last step is complex [63]. P450 17A1 is complicated. Although there is general agreement that the first step, the 17α-hydroxylation, involves a classical Compound I reaction, the second step has been proposed to involve either a Compound 0 or Compound I reaction [51,85,86,88,92,181]. Unfortunately, the mechanism of cleavage of α-ketols cannot be unambiguously resolved with the ^18^O_2_ labeling method [85,86,200]. The incorporation of ^18^O into the formic acid approach has been used with both P450 19A1 and 51A1. In both cases, deformylation occurs (to generate formic acid). With P450 19A1 the third step now appears to involve mainly Compound 0 (measured > 90% Compound 0, <1% Compound I) [195], but with P450 51A1, there is a mixed mechanism with the contributions of both Compound 0 and Compound I mechanisms (measured 88% Compound 0, 14% Compound I), plus a Baeyer–Villiger rearrangement, depending on the enzyme [202]. The chemistry of these different courses is undoubtedly dictated by elements of the different proteins, although it is not yet clear exactly what these are.

**Figure 41:**
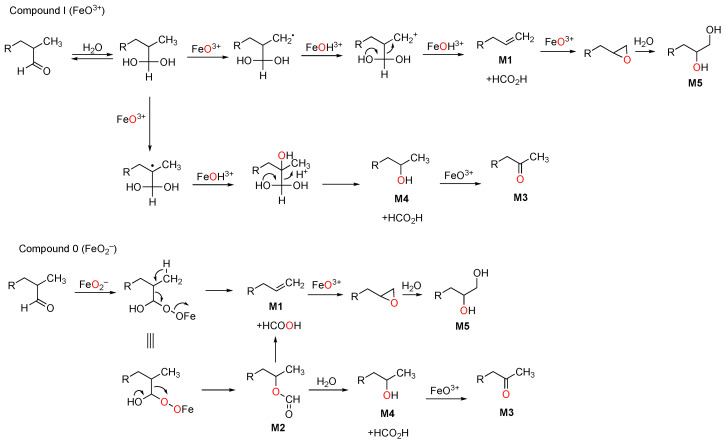
Oxidative deformylation of an intermediate in cholesterol metabolism by P450 125A1 [239]. The red oxygen atoms indicate the course of tracking through the proposed mechanisms.

Although the ^18^O labeling approach is not unambiguous regarding the chemical mechanism of catalysis involving α-ketols, there are still other reactions where this approach could be applied. One is with some of the reactions of bacterial P450 125A1 (Figure 41) [239,240]. In that work, the authors considered products derived from the deformylation of 26-oxocholesterol (aldehyde at one of the two methyl carbons on the sterol tail). Although formic acid is assumed to be a product, it was not documented. A reservation about the conclusions is that the ^18^O-labeling experiments were done with (*d*_7_) chloest-4-ene-3-one as the substrate and not the aldehyde intermediate. Thus, the initial C26 hydroxylation will have ^18^O incorporated, which will be present in the 26-aldehyde. That oxygen may or may not exchange with the H_2_^16^O solvent prior to further oxidation, depending on the kinetic processivity of the reaction. The authors were not actually sure (footnote *a* of the Table 1 of that publication). Only some of the products are analyzed for ^18^O (Table 1 and Scheme 2 of that publication) and not formic acid.

Accordingly, no ^18^O analysis of formic acid was conducted. Formic acid, if released in the enzyme reaction, would not be expected to be sequestered near the putative carbocation (that would be formed by its release) and then react to form the Baeyer–Villiger product. The mechanism is proposed to involve the capture of the carbocation by water (or hydroxide) to generate the product M4. The trapping of formate seems unlikely. The authors do raise the possibility that a (stable) Baeyer–Villiger product (M2) is formed. The evidence for this structure is limited to a parent ion in the mass spectrum (full scan not shown) and possibly confounded by peak overlap with the aldehyde and the deuteration. Nevertheless, this may be a Baeyer–Villiger product. Its stability was not examined. In addition to the pathway shown in Figure 41, M2 could hydrolyze to give the alcohol M4 or be eliminated to form the olefin M1.

**Figure 42:**
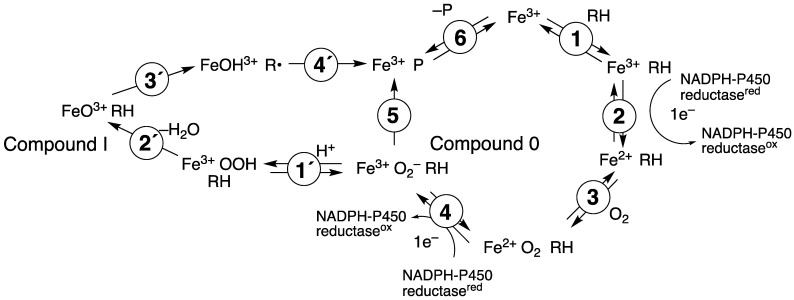
Rationalization of chemical mechanisms of catalysis of aldehyde intermediates in steroid metabolism [195,202].

The current view of the mechanisms of P450 19A1 and 51A enzymes is that shown in Figure 42, with the usual P450 catalytic cycle (Figure 2) split into two sections. Thus, none of the oxidations we have studied involve Compound 0 exclusively. The Compound 0 cycle consists of Steps 1–6. At the stage of Compound 0 (Fe^3+^O_2_^–^ RH), competition exists between protonation (Step 1′) and the nucleophilic attack of an aldehyde (Step 5). The attack on the aldehyde predominates in the cases of P450 19A1 and 51A1 (at least with their preferred substrates), but apparently some of the complex is protonated (Step 1′) and goes on to Compound I (FeO^3+^). Compound I can carry out these same reactions. Thus, in an experiment where Compound I is generated artificially (e.g., Zhang et al. [188]), some product is formed.

With human P450 51A1, we worked with a site-directed mutant (D213A) that was designed to attenuate protonation [222] (Step 1′) and found some decrease in the ^18^O labeling of formic acid from H_2_^18^O (i.e., 14-formyl ^18^O dihydrolanosterol) [202], but there is difficulty in designing site-directed mutants that will be more likely to generate Compound 0 from protonation than in the wild-type enzyme. It appears that these aldehydes are uniquely poised to react with the nucleophilic oxygen anion (of Compound 0), as suggested by the geometry in the X-ray structure of human P450 51A1 (Figure 37) [202]. One explanation is that these deformylating enzymes are biologically very important and also optimized for attack of the aldehyde (see Figure 27 and Figure 33), but we have conducted similar ^18^O labeling studies with rabbit P450 2B4 and ODEC [195] plus some very simple aldehydes [201] and also find high contents of ^18^O incorporated (from ^18^O_2_) into formic acid, arguing against tight coupling. Apparently, the Compound 0 forms of these enzymes (P450s 2B4, 19A1, and 51A1) all react rapidly with formyl carbonyls. What we do not know is whether (i) this reactivity extends to other electrophilic groups (e.g., imines, nitriles) or (ii) what can happen with some ketones. Many aldehydes are also oxidized to carboxylic acids (including 19-oxo androstenedione and 19-oxo testosterone [170] and ODEC [195]), presumably involving a Compound I mechanism (i.e., abstraction of a hydrogen atom to form a *gem*-diol or the aldehyde itself).

## Data Availability

All data are included in the manuscript or in the cited references.
